# Evaluation via Supervised Machine Learning of the Broiler Pectoralis Major and Liver Transcriptome in Association With the Muscle Myopathy Wooden Breast

**DOI:** 10.3389/fphys.2020.00101

**Published:** 2020-02-25

**Authors:** Chelsea A. Phillips, Benjamin J. Reading, Matthew Livingston, Kimberly Livingston, Chris M. Ashwell

**Affiliations:** ^1^Prestage Department of Poultry Science, North Carolina State University, Raleigh, NC, United States; ^2^Department of Applied Ecology, North Carolina State University, Raleigh, NC, United States

**Keywords:** wooden breast, machine learning, poultry transcriptomics, support vector machines, artificial neural networks, transforming growth factor

## Abstract

The muscle myopathy wooden breast (WB) has recently appeared in broiler production and has a negative impact on meat quality. WB is described as hard/firm consistency found within the pectoralis major (PM). In the present study, we use machine learning from our PM and liver transcriptome dataset to capture the complex relationships that are not typically revealed by traditional statistical methods. Gene expression data was evaluated between the PM and liver of birds with WB and those that were normal. Two separate machine learning algorithms were performed to analyze the data set including the sequential minimal optimization (SMO) of support vector machines (SVMs) and Multilayer Perceptron (MLP) Artificial Neural Network (ANN). Machine learning algorithms were compared to identify genes within a gene expression data set of approximately 16,000 genes for both liver and PM, which can be correctly classified from birds with or without WB. The performance of both machine learning algorithms SMO and MLP was determined using percent correct classification during the cross-validations. By evaluating the WB transcriptome datasets by 5× cross-validation using ANNs, the expression of nine genes ranked based on Shannon Entropy (Information Gain) from PM were able to correctly classify if the individual bird was normal or exhibited WB 100% of the time. These top nine genes were all protein coding and potential biomarkers. When PM gene expression data were evaluated between normal birds and those with WB using SVMs they were correctly classified 95% of the time using 450 of the top genes sorted ranked based on Shannon Entropy (Information Gain) as a preprocessing step. When evaluating the 450 attributes that were 95% correctly classified using SVMs through Ingenuity Pathway Analysis (IPA) there was an overlap in top genes identified through MLP. This analysis allowed the identification of critical transcriptional responses for the first time in both liver and muscle during the onset of WB. The information provided has revealed many molecules and pathways making up a complex molecular mechanism involved with the progression of wooden breast and suggests that the etiology of the myopathy is not limited to activity in the muscle alone, but is an altered systemic pathology.

## Introduction

The occurrence of wooden breast (WB) in commercial poultry production is rising, leaving producers with an inferior product and, ultimately, unsatisfied consumers (Mudalal et al., 2014; Petracci et al., 2015). Much of the incidence is thought to be attributed to artificial selection that has led to the development of broilers with greater muscle yield, better feed conversion rates, and faster growth. Frequent detection of muscle myopathies has been thought to be associated with increased growth rates and breast muscle yields (Sihvo et al., 2014; Trocino et al., 2015; Kuttappan et al., 2016; Abasht et al., 2019). WB is described as a hard or firm consistency deep within the pectoralis major (PM) muscle (Sihvo et al., 2014). Alterations of the meat composition have been observed including increased moisture, collagen, sodium, calcium and fat content (Zambonelli et al., 2014). When compared to PM without the myopathy, the PM meat quality with WB is lower due to greater cooking losses and greater shear force when compared to PM without the myopathy (Zambonelli et al., 2014; Trocino et al., 2015). Consequently, the meat texture of the breast is tougher and less desirable to the consumer. Extensive histological evaluation of WB has been characterized by necrosis, chronic fibrosis, infiltration of fat and connective tissue, and the presence of inflammatory cells and macrophages (Sihvo et al., 2014; Trocino et al., 2015; Kuttappan et al., 2016; Papah et al., 2017).

The severity of WB is often categorized on a scale ranging from 0 to 3 (0, normal; 1, mild; 2, moderate; 3, severe) (Trocino et al., 2015). WB lesions can be detected through manual palpation of the PM as early as 3 weeks of age (Mutryn et al., 2015). Research covering muscle myopathies in broilers reveals that myopathies have increased in recent years and have been correlated with the selection of larger breast muscle (Petracci et al., 2015). The cause of WB is currently unknown, but it is more prevalent in older, heavier male broilers than young birds (Kuttappan et al., 2017; Brothers et al., 2019). Larger breast muscle has been associated with hypertrophied muscle fibers which is thought to impact blood supply and number of satellite cells that are needed in muscle regeneration (Daughtry et al., 2017; Malila et al., 2019).

The increase in prevalence and severity of WB has the potential to result in excessive economic loses. It has been projected that these losses could exceed more than $200 million USD/yr (Kuttappan et al., 2016). Often, the PM muscle of birds with WB can appear pale, bulging, and covered in a clear viscous fluid (Sihvo et al., 2014). Considering consumers frequently purchase chicken breasts based on visual appearance of the meat, this unpleasant appearance will undoubtedly have a negative impact on sales (Petracci et al., 2015). Even if WB is undetectable by the outward appearance prior to purchase, the hardness of the inner muscle will become evident once the meat is handled or consumed. Ultimately, this is bad for the consumer, the company, and poultry production as a whole by breaking consumer trust and potentially initiating the desire for an alternative product.

Currently, researchers are investigating the nutritional, physiological, and genetic factors that surround this myopathy through the use of many common techniques that span from the inclusion of feed additives to molecular approaches using gene expression analysis and histology (Velleman and Clark, 2015; Kuttappan et al., 2016; Papah et al., 2018; Petracci et al., 2019). In the present study, we use machine learning analysis of PM and liver transcriptome datasets to capture the complex relationships that are not typically revealed by traditional statistical methods. This was achieved through the use of algorithms to identify genes within an extensive RNA sequencing dataset whose expression can be used to correctly distinguish normal tissue apart from severe/moderate WB. Previous gene expression datasets have characterized many molecular relationships present in birds with WB (Mutryn et al., 2015; Papah et al., 2018; Brothers et al., 2019). This study uses a different mechanism to evaluate gene expression data in the hopes for a more concise evaluation of the WB myopathy.

## Materials and Methods

### Facilities and Rearing

This experiment was conducted at the North Carolina State University Chicken Education Unit between the months of March and April, 2019. All procedures used in this study were reviewed and approved by the Institutional Animal Care and Use Committee. Eggs were collected from a resident 25-week-old broiler breeder flock of known, similar genetic background and stored for no more than 7 days at 15°C. Incubation was performed based on the methods described by Livingston (Livingston et al., 2018a). Day of hatch chicks were sex-sorted and a total of 128 male chicks were individually neck tagged and placed into eight replicate pens (1.2 m × 4.0 m; 4.8 m^2^) with 16 chicks per pen (blocked by location within the house). Each pen was supplied with one bell water drinker, two tube feeders, and bedded with fresh pine shavings (15 cm deep). Broilers were provided *ad libitum* access to a common commercial starter diet (1–14 days) and a common grower diet (14–45 days) manufactured at the NC State University Feed Mill (Table 1). Eight broilers from each pen, for a total of 64, were selected (based on experiment-wise mean BW) for processing at 45 days. Birds were evaluated for WB and samples of PM and liver tissue were collected for analysis of gene expression and stored at −80°C. PM tissue was sampled from the deep medial region of the birds left breast and liver tissue was sampled from caudal region of the left lobe.

**TABLE 1:** Composition of basal starter and grower diets^1^.

**Ingredients**	**Starter**	**Grower**
Corn	55.22	57.45
Soybean meal (48% CP)	36.9	31.87
Poultry fat	2.36	5
Dicalcium phosphate (18.5% P)	2.02	2.39
Glycine	1.25	1.25
Limestone	0.77	0.6
Salt	0.5	0.5
DL-Methionine	0.28	0.24
Choline chloride (60%)	0.2	0.2
L-Threonine	0.1	0.09
L-Lysine	0.05	0.07
Selenium premix^2^	0.05	0.05
Vitamin premix^3^	0.05	0.05
Mineral premix^4^	0.2	0.2
Coccidiostat^5^	0.05	0.05
**Total**	**100**	**100**
Calculated nutrient content		
Crude protein	22.5	20.2
Calcium	0.9	0.9
Available phosphorus	0.45	0.5
Potassium	0.89	0.82
Total lysine	1.27	1.14
Total methionine	0.62	0.55
Total threonine	0.85	0.76
Total methionine + cysteine	0.97	0.87
Sodium	0.21	0.2
Metabolizable energy (kcal/g)	2.85	3.03

*^1^Starter diet was fed to approximately 14 days of age, 1820 g per bird. ^2^Selenium premix provided 0.2 mg Se (as Na_2_SeO_3_) per kg of diet. ^3^Vitamin premix supplied the following per kg of diet: 13,200 IU vitamin A, 4,000 IU vitamin D_3_, 33 IU vitamin E, 0.02 mg vitamin B_12_, 0.13 mg biotin, 2 mg menadione (K_3_), 2 mg thiamine, 6.6 mg riboflavin, 11 mg d-pantothenic acid, 4 mg vitamin B_6_, 55 mg niacin, and 1.1 mg folic acid. ^4^Mineral premix supplied the following per kg of diet: manganese, 120 mg; zinc, 120 mg; iron, 80 mg; copper, 10 mg; iodine, 2.5 mg; and cobalt, 1 mg. ^5^Coccidiostat supplied monensin sodium at 90 mg/kg of food.*

### Processing

At 45 days, selected broilers were collected and transported to the North Carolina State University Chicken Education Unit’s broiler processing facility followed by shackling and stunning in a salt saturated saline head stun cabinet. Birds were head stunned with a 110v/60hz CF2000 poultry stun knife set to 150 mA for 10 s. Broilers were exsanguinated for 120 s by opening of the jugular vein and carotid artery with a single knife cut by a trained technician followed by scalding in hot water (60°C) for 120 s. This was followed by feather picking for 30 s (Meyn Food Processing Technology B.V., Westeinde Amsterdam, Netherlands). Head and feet were removed, vent opened (VC Poultry Vent Cutter, Jarvis Product Corp., Middleton, CT, United States), and viscera and giblets removed manually. Liver and PM tissue were removed and snap frozen on liquid nitrogen for RNA sequencing analysis. Hot carcass weights (HCW) were collected prior to carcasses being air chilled at 3.0°C for approximately 24 h. At 24 h postmortem examination of the PM muscle was evaluated after breast muscle was removed from carcass and bones by a trained and experienced technician for WB using a one to four-point ordinal scale of measurement in accordance with the methods described previously by Livingston with a score of one being normal and four being most severe (Livingston et al., 2018b).

### RNA Sequencing Analysis

Pectoralis major and liver tissue samples from 45 d broilers with varying severities of WB scores were obtained and preserved in RNALater. RNA extracted from the PM of 15 birds with moderate to severe WB was compared to RNA extracted from five normal PM. For liver RNA evaluation, 10 samples from birds with moderate to severe WB and five livers from birds with normal PM were used. RNA was extracted from the tissues using Qiagen RNeasy Mini protocol (Qiagen, Valencia, CA, United States) following the manufacturer’s instructions. The RNA quality was assessed by Nanodrop 2000 spectrophotometer (Thermo, United States). Two micrograms of RNA from each sample were taken to the North Carolina State University Genomics Sciences Laboratory for library preparation and sequencing on the Illumina HiSeq 2500 sequencer. RNA sequencing was analyzed using CLC Genomics Workbench (Qiagen, Valencia, CA, United States; licensed to NCSU) version 11 following the software manual^1^. RNA sequencing reads and annotations were mapped to the chicken genome (*galgal5*) from NCBI. Raw reads were processed by the default settings of reads’ quality control and adapter trimming. The false discovery rate p-value (FDRp) was calculated to correct for multiple testings’ and an FDR adjusted *p* ≤ 0.05 was considered statistically significant. Fold change and Log_2_ fold change differences in gene expression between WB scores moderate to severe and normal were also calculated.

### Machine Learning Analysis

Gene expression data were analyzed from the PM and liver using the Waikato Environment for Knowledge Analysis (WEKA) version 3.8.3. Two different pattern recognition machine learning algorithms were performed to analyze the data set: sequential minimal optimization support vector machines (SVMs) and artificial neural network multilayer perceptron (MLP) (Cortes and Vapnik, 1995; Eibe et al., 2016). The machine learning algorithms were compared to identify gene expression patterns within the data set of 15,569 genes, which could be used to correctly classify birds as either exhibiting moderate to severe WB or normal (dichotomous class assignment). Briefly, the 15,569 genes from the gene expression dataset were ranked based on Shannon Entropy (Information Gain) in dichotomous classification assignment by SVMs (Keerthi et al., 2001; Eibe et al., 2016). Information gain ranking was then used to identify those gene expression patterns most relevant to assignment of each bird as having WB or normal by either MLP or SVMs. Reduction of data dimensionality for each machine learning algorithm was then performed by sequential exclusion of those gene expression patterns least relevant to the class assignment (50–2,000 per sequence). This step eliminates overfitting of the machine learning classifiers. To identify the minimum number of gene expression patterns required for classification, 1–50 genes were sequentially excluded from the dataset until only the top 2–7 remained. This step identifies underfitting of the machine learning classifiers and the point of optimal classification for the MLP and SVMs was determined to be the intersection between the underfitting and overfitting curve plots.

Class assignment of all machine learning algorithms was evaluated *vis-à-vis* by two cross-validation strategies (classification as either WB or normal). The first being a percentage split, where 66% of the total data were randomly used for training and the remaining 34% of the data were used in testing. The second cross-validation was a stratified hold-out (*n*-fold) method with 5-fold, where 4-fold of the randomized gene expression data were used for the training and 1-fold was used for testing. This was repeated five times, such that all normal replicate samples were used at least once in testing and the average model performance was recorded.

The performance of the two machine learning algorithms SVMs and MLP was determined using percent correct classification during the cross-validations, which indicated the likelihood that each individual biological replicate could accurately be assigned into the classes of WB or normal based on the gene expression data provided. Kappa statistic and ROC score also were recorded. Any kappa statistic greater than 0 indicated that the machine learning classifier is performing better than random chance along with a ROC score of greater than 0.500. The random probability of chance for dichotomous assignment was assumed to be 50% based on the Law of Probability.

A negative control of machine learning was created through 10 separate randomizations of the individual birds within the dataset. The SVMs and MLP were unable to predict WB or normal, indicating the machine learning herein is true.

### Pathway Analysis

Ingenuity Pathway Analysis (IPA; Qiagen, Valencia, CA, United States)^2^ software was used for canonical pathway analysis, upstream regulatory analysis, and gene network discovery. SVM analysis of the top 450 performing differentially expressed genes from the PM of birds with moderate/severe to normal WB were used in IPA and the top 150 were used from the liver dataset. IPA calculation of z-scores using the gene expression fold change values measures the state of activation or inhibition of the molecules involved in the molecular networks. The analysis of biological mechanisms occurring in the differentially expressed genes of the chicken in IPA are based on mammalian systems for human, rat, and mouse.

## Results

### Multilayer Perceptron

**FIGURE 1:**
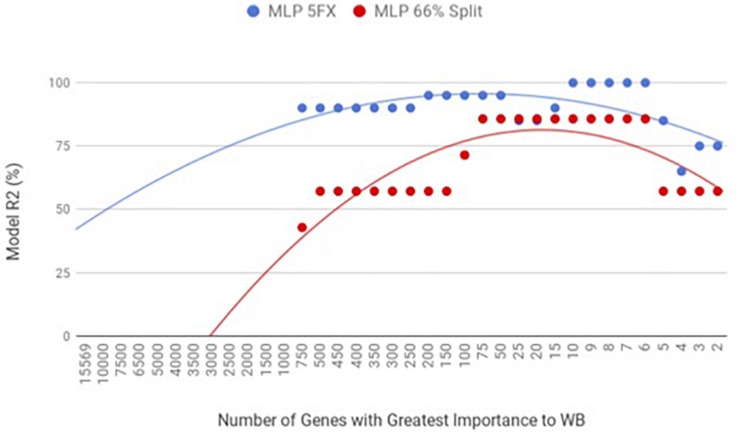
WB PM transcriptome dataset by 5x cross-validation and 66% percent split using Multilayer Perceptron (MLP).

**FIGURE 2:**
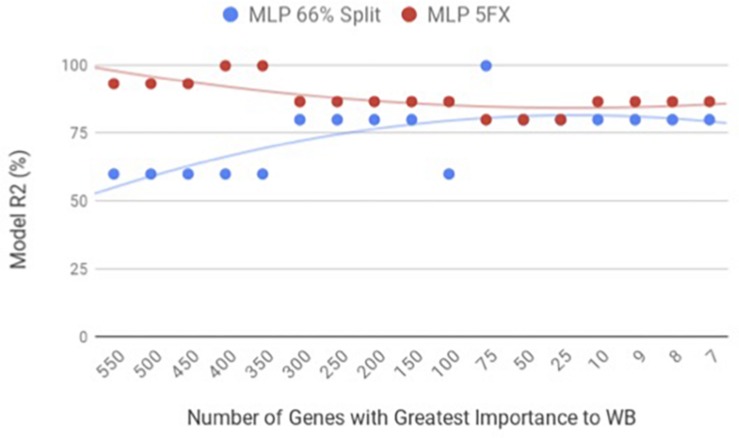
WB Liver transcriptome dataset by 5x cross-validation and 66% percent split using Multilayer Perceptron (MLP).

By using the novel approach of evaluating the WB PM transcriptome dataset by 5-fold cross-validation using MLP, the expression of nine genes (*NUP43, KPNA7, DEAF1, NUD19, CCDC85A, SLC25A30, ENSGAL00000015075, PACSIN3*, and *RPL19*) were able to correctly classify if the PM tissue from an individual bird was normal or exhibited WB in 100% of the individual genomes when using the top nine genes ranked based on Shannon Entropy (Information Gain) (Figure 1). MLP is an artificial neural network, which can distinguish data that are not linearly separable, but instead is a feed forward mechanism that maps a dataset into suitable outputs. The top nine genes in the PM were further analyzed for their individual biological roles (Table 2). When the liver transcriptome dataset was evaluated by 66% split using MLP, the expression of 75 genes were capable of correctly classifying the PM tissue of an individual bird as exhibiting WB or normal in 100% of the individual genomes (Figure 2). The kappa statistic and ROC score were optimal at 1 for both PM and liver. The individual expression of transcripts *CARD19* and *ITCH* predicted WB or normal using MLP 5-fold cross validation 93.333% of the time whereas *BUD13* and *ENSGALG00000039590* individually predicted WB or normal 100% of the time using 66% split (Table 3).

**TABLE 2:** Top nine regulators of differential expressed genes in the PM of birds with normal PM compared to severe/moderate WB by 5-fold cross-validation with 100% correct classification and percent split with 85.71% correct classification using MLP.

**Gene**	**Gene type**	**Short description**	**Log-fold change**	***P*-value**
*NUP43*	Protein Coding	Forms Nuclear Pore Complex (NPC)	0.2005	0.4900
*KPNA7*	Protein Coding	Forms Nuclear Pore Complex (NPC)	−3.4539	2.62E-06
*DEAF1*	Protein Coding	Zinc Finger Domain Transcription Regulator; Inhibits Cell Proliferation	0.3406	0.2972
*NUDT19*	Protein Coding	Enzyme involved in Peroxisomal Lipid Metabolism	0.9608	1.28E-04
*CCDC85A*	Protein Coding	Unknown	2.0774	0.0009
*SLC25A30*	Protein Coding	Renal Mitochondrial Carrier	0.1223	0.9574
*ENSGALG00000015075*	Protein Coding	Beta-1,3-glucuronyltransferase 1	−0.3370	0.5767
*PACSIN3*	Protein Coding	Links Actin Cytoskeleton with Vesicle Formation	−0.9920	1.58E-04
*RPL19*	Protein Coding	Ribosomal Protein Component of the 60S Subunit	−0.8323	0.0066

**TABLE 3:** Top regulators of differential expressed genes in the liver of birds with normal PM compared to severe/moderate WB tissues using MLP.

**Gene and Validation Method**	**Gene Type**	**Short Description**	**Log-fold change**	***P*-value**
***CARD19*** *Stratified hold-out (93.333%)*	Protein Coding	Caspase Recruitment Domain Family Member 19; Negative Regulation of IKKß/NFKB Cascade; Regulation of Apoptosis	−2.5547	0.2831
***ITCH*** *Stratified hold-out (93.333%)*	Protein Coding	Related pathways are signaling by Sonic Hedgehog and TNF signaling pathway. Plays a role in erythroid and lymphoid cell differentiation and regulation of immune responses.	−1.2210	0.7238
***BUD13*** *Percent split (100%)*	Protein Coding	Component of the active spliceosome involved in pre-mRNA splicing	1.2022	0.6601
***ENSGALG00000039590*** *Stratified Hold-Out (93.333%) and Percent Split (100%)*	Protein Coding	Unknown	−2.0511	0.1209

### Sequential Minimal Optimization

**FIGURE 3:**
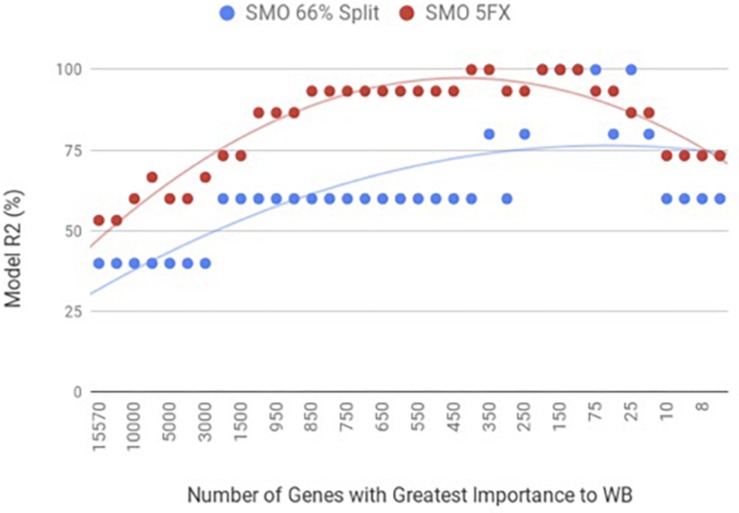
WB PM transcriptome dataset by 5x cross-validation and 66% percent split using Sequential Minimal Optimization (SMO).

**FIGURE 4:**
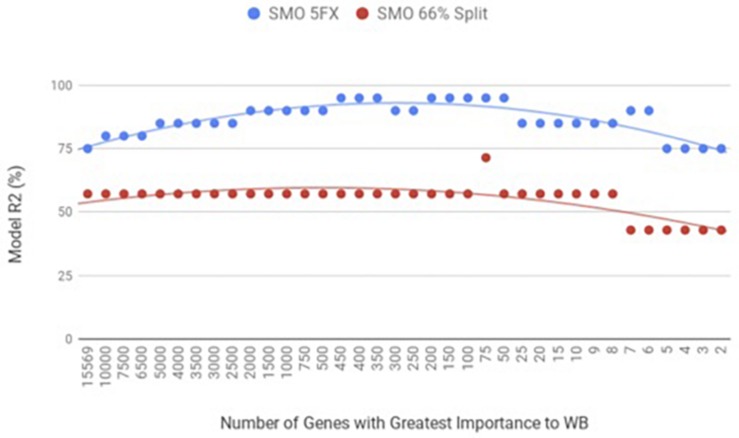
WB Liver transcriptome dataset by 5x cross-validation and 66% percent split using Sequential Minimal Optimization (SMO).

When the PM gene expression data set was evaluated between birds with WB and those that were normal using WEKA SMO function of SVMs by a 5-fold cross-validation method they were correctly classified 95% of the time using 450 of the top genes ranked based on Shannon Entropy (Information Gain) as a preprocessing step (Figure 3). The kappa statistic and ROC score were optimal at 0.8571 and 0.9000, consecutively. The liver gene expression data set was evaluated using the SMO function of SVMs by both a 5-fold cross-validation method and the 66% split method was capable of predicting WB or normal 100% of the time with 100 to 200 of the top genes ranked based on Shannon Entropy (Information Gain) (Figure 4). Optimal kappa statistic and ROC scores of 1 were achieved for both methods. In both machine learning algorithms, the stratified hold-out method appeared to accurately estimate the machine learning classifier correctly more often than the percentage split method.

### Ingenuity Pathway Analysis

Lastly, Ingenuity Pathway Analysis (IPA) was used to evaluate the top 450 genes ranked based on Shannon Entropy (Information Gain) from the PM transcriptome using SMO and 150 of the top genes ranked based on Shannon Entropy (Information Gain) for the liver transcriptome using SMO. Interestingly, many of the top analysis ready molecules detected in IPA were the same molecules detected using the completely separate machine learning approach of MLP. The repeatability in identification of these genes leads to greater confidence in the role they are having in the WB myopathy. These included *BUD13* from the liver transcriptome dataset and *CCDC85A* and *KPNA7* from the PM dataset (Tables 2, 3).

**FIGURE 5:**
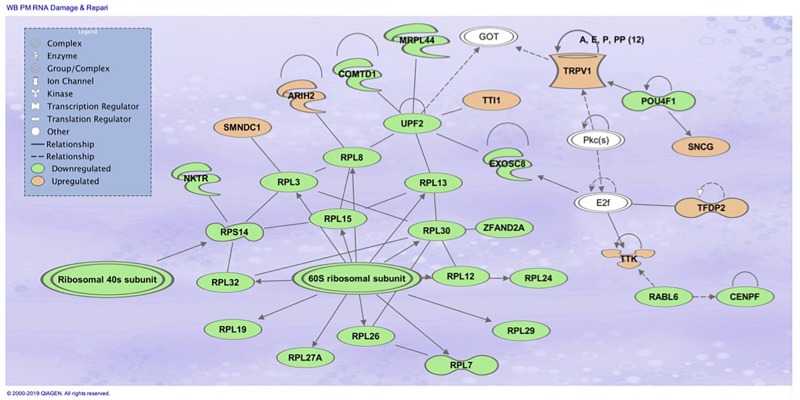
Top associated network function in IPA for the PM transcriptome dataset of birds with normal PM compared to severe/moderate WB: *RNA Damage and Repair and Protein Synthesis*.

**FIGURE 6:**
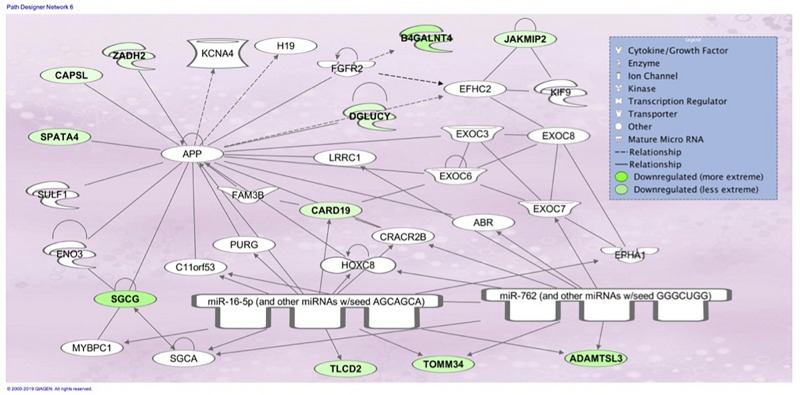
Associated network function in IPA for the liver transcriptome dataset of birds with normal PM compared to severe/moderate WB: *Skeletal and Muscular System Development and Function, Developmental Disorder, Hereditary Disorder*.

**FIGURE 7:**
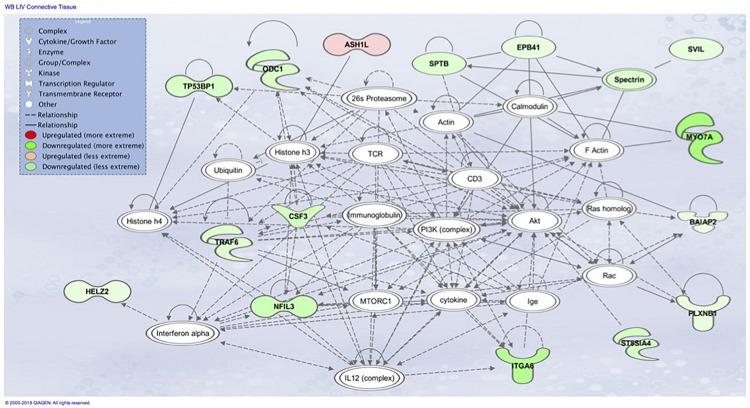
Associated network function in IPA for the liver transcriptome dataset of birds with normal PM compared to severe/moderate WB: *Connective Tissue Disorders, Hematological Disease, Hereditary Disorder*.

**FIGURE 8:**
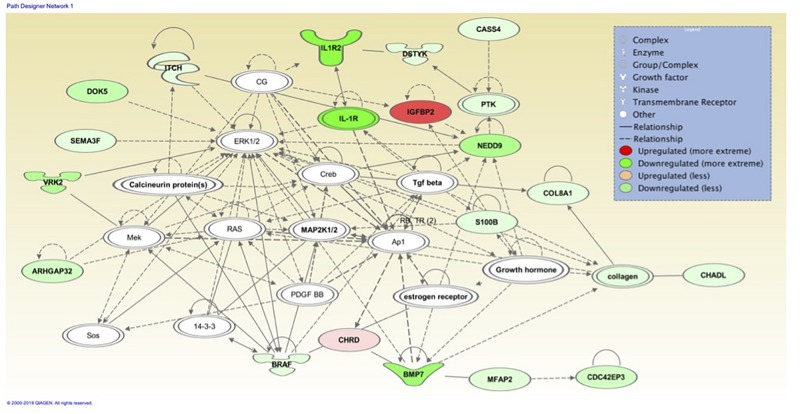
Associated network function in IPA for the liver transcriptome dataset of birds with normal PM compared to severe/moderate WB: *Cell Cycle, Embryonic Development, Cellular Movement.*

The top associated network function in IPA for the PM transcriptome dataset was for RNA Damage and Repair, Protein Synthesis. This pathway involves *RPL19* which is a component of the 60S ribosomal subunit detected through MLP of WEKA (Figure 5). When evaluating the liver transcriptome dataset three top network associations identified were Skeletal and Muscular System Development and Function, Developmental Disorder, Hereditary Disorder (Figure 6), Connective Tissue Disorders, Hematological Disease, Hereditary Disorder (Figure 7), and Cell Cycle, Embryonic Development, Cellular Movement (Figure 8). Figure 6 depicts *CARD19* which was also detected using MLP of WEKA. This network is an association of the relationship this group of molecules has in association with the skeletal and muscular system development and function. Figure 8 represents molecules in the cell cycle such as *TGF-β*, *MAP2K1/2*, and calcineurin proteins which are important in skeletal muscle myoblast regulation and differentiation.

When comparing the performance of the top ranked genes between MLP and SVM, many genes were identified as top performing in both ML models. In the PM data set, evaluation of the top nine molecules with increased expression, *KPNA7* was observed in both MLP (100% classification) and the top analysis ready molecules in IPA from the best performing data set using SVM (Table 2). *RPL19* was observed as a top regulator in MLP with the ability to correctly classify WB or normal 100% of the time and was an affected molecule in the IPA analysis of the top associated network (Figure 5). In the same data set *CCDC85A* was observed as a molecule with decreased expression in both ML models and able to correctly classify WB 100% of the time using MLP (Table 2).

Assessing the liver dataset there were many overlaps using the two ML models. *BUD13* was a top regulator in MLP and a top analysis ready molecule with increased expression in IPA (Table 3). The associated network related to skeletal and muscular system development and function from IPA had *CARD19* as a downregulated molecule in IPA and a top regulator in MLP that was able to predict WB or normal 93.33% of the time (Figure 6). Lastly, the IPA network associated with cell cycle, embryonic development, and cellular movement included the down regulation of the *ITCH* molecule which was also detected in MLP as being able to correctly classify 93.33% of the time (Figure 8).

## Discussion

Prior systematic studies of the abnormal muscle phenotype referred to as wooden breast using gene expression measurements has identified multiple processes that may contribute to the development of the myopathy. Processes including muscle fiber regeneration, inflammatory response, myodegeneration, hypoxia, fibrosis, lipidosis, and altered energy metabolism are likely involved in the manifestation of wooden breast (Mutryn et al., 2015; Zambonelli et al., 2016; Papah et al., 2018; Malila et al., 2019; Petracci et al., 2019). This study attempts to connect organismal level physiology and metabolism with the activity in the breast muscle by including the evaluation of liver gene expression by a novel analysis method.

### Machine Learning Approach

Traditional statistics is not well designed to handle datasets which have more variables than observations therefore this is an alternative method to analyze and make interpretations of datasets. Using the traditional statistical approach on this dataset resulted in almost 1000 genes being identified as differentially expressed (FDR corrected *p* < 0.05) across the etiology of WB in the PM and only one gene in the liver. This large number of targets (or too few) when subjected to pathway analysis in IPA results in more pathways (or none) than can be interpreted, therefore an alternative approach was warranted. The use of machine learning on the transcriptome datasets allows for the recognition of consistent patterns or systematic relationships within the datasets and therefore can be used to make predictions. Through this process, the machine learns by building a model from example inputs and then makes predictions on new data by the learned pattern recognition. This is the first report using machine learning to identify gene expression patterns associated with WB in muscle as well as peripheral tissues that may be influencing the myopathy development.

### PM Fibrosis

Evaluating the top nine genes ranked based on Shannon Entropy (Information Gain), all the genes are protein coding genes (Table 2). The top two genes *NUP43* and *KPNA7* are two essential Nuclear Pore Complexes (NPC). *KPNA7* was also observed as one of the top nine upregulated genes with a fold-change for birds with WB (Mutryn et al., 2015). NPC’s are macromolecular proteins found within the nuclear envelope in eukaryotic cells. These complexes are surrounded by decondensed chromatin and are responsible for the exchange of large molecules such as proteins and RNA between the nucleus and cytoplasm (Kelley et al., 2010). Prior to cell division there is an increase in expression of NPC found in the nuclear envelope (NE). Once mitosis begins the NE is broken down and NPCs form subcomplexes, which are essential for later reassembly of the NPC. This process of reconstruction is unknown, however, it has been shown when the KPNA7 NPC is depleted, mitotic defects and deformation of the nucleus occur (Vuorinen et al., 2018). In the mouse, *KPNA7* is mostly expressed in oocytes and zygotes and is responsible for epigenetic reprogramming which occurs during fertilization and zygotic gene activation (Hu et al., 2010). Recently, NPCs have been observed in regulation of gene expression and have been associated with both gene silencing and activation (Galy et al., 2000; Casolari et al., 2004). The expression of nucleoporins, which make up the NPCs, vary depending on cell types and changes in the structure of NPCs are used in the regulation of cellular differentiation (D’Angelo, 2018). In mammals, the tissue specific NPC, Nup210 has an effect on the regulation muscle development and maintenance. Nup210 regulates myofiber maturation, growth and even survival through the use of a dependent transcription factor Mef2C in the regulation of structural and maturation related muscle genes (D’Angelo et al., 2012; Raices et al., 2017). In the absences of Nup210 the initial formation of muscle fibers occurs during development, however in older animals abnormal muscle structure develops and muscle degeneration can even occur (Raices et al., 2017).

### PM Regeneration

Another top gene, *DEAF1*, is a part of the SAND domain in the molecule Ski. The activity of Ski was originally identified in the chicken as a transduced retroviral oncogene, however, research has indicated homologs which are not associated with endogenous viral loci (Li et al., 1986). The c-Ski residue is primarily found in the nucleus and is highly conserved in many species. The SAND domain is involved in protein-protein interactions and is responsible for the interactions of SKI with Smad4, FHL2, and MeCP2 (Engle et al., 2008). Ski can act as an activator or repressor to gene transcription depending on the transcription factor it interacts with. C-Ski has been shown to bind with Smad4 and block activation of transforming growth factor (TGF-β). TGF-β leads to an increase in β-catenin within the cytoplasm and β-catenin is an activator of canonical Wnt signaling (Staal, 2016). TGF-β, TNF-α, and IGF-2 are growth factors that regulate myoblast differentiation rather than activation (Rosenthal et al., 1991; Li et al., 2005; Carter et al., 2009). The key role of TGF-β in muscle repair is to regulate the balance between fibrosis and regeneration. In Japanese quail, myogenic differentiation is induced through the activation of myogenic regulatory factors (MRFs), MyoD, and myogenin as well as inhibiting HDAC activity (Colmenares and Stavnezer, 1989; Kobayashi et al., 2007). Satellite cells are activated by expression of early myogenic regulatory factors, MyoD and Myf5. Later, myogenin and MRF4 are expressed. The Ski response element to myogenin is located upstream of the promoter region.

The gene RPL19 was discovered as one of the top nine regulators in the MLP model able to 100% correctly classify PM samples as WB or normal sample. This protein coding gene encodes a ribosomal protein that makes up the 60S ribosomal subunit (Davies and Fried, 1995). Like many of the genes observed, RPL19 also plays a role in genetic regulation. In mammals, this protein contains a CpG island at the 5′ transcriptional start site, which would indicate an area for modifications to the expression of this gene (Davies and Fried, 1995). RPL19 was also involved in the top molecular network associated with RNA damage and repair protein synthesis when evaluating the 450 attributes that were 95% correctly classified using SMO through Ingenuity Pathway Analysis (IPA) (Figure 5). Transcripts related to spliceosomes were also detected in IPA analysis in Figure 6, involving the previously mentioned gene *BUD13* as a component of the spliceosome. Similar to this finding previous research evaluating gene expression data in the PM has indicated differential expression of small nucleolar RNAs including snoRNAs and miRNAs, which are often involved in ribosomal and protein synthesis (Zambonelli et al., 2014).

*CCDC85A*, another top regulator identified with MLP, was able to correctly classify as a single attribute WB and normal PM birds 95% of the time using stratified cross-validation. *CCDC85A* was also a top analysis molecule when evaluating the 450 attributes that were 95% correctly classified using SMO through Ingenuity Pathway Analysis (IPA). *CCDC85A* is a protein coding gene for coiled coil domain containing 85a (Iwai et al., 2008). In humans, *CCDC85A* is regulated by p53 and results in the degradation of β-catenin. This protein suppresses β-catenin activity through interaction with T-cell factors to result in Wnt signaling (Iwai et al., 2008). β-catenin is an activator of canonical Wnt signaling (Staal, 2016). Wnt signaling is responsible for the activation of satellite cells in adult skeletal muscle and perturbations of this pathway can result in muscle fibrosis (Cisternas et al., 2014). If Wnt signaling occurs too often, the satellite cells become exhausted and eventually lose the ability to renew (Ryall et al., 2008). This has been characterized by increased extracellular matrix molecules such as fibronectin, collagen, and macrophages leading to the inability of muscles to regenerate and ultimately the loss of activity, leading to similar traits associated with WB (Cisternas et al., 2014). The addition of Wnt3A protein in mice has been shown to increase the rate at which progenitor cells are converted from a myogenic to a fibrogenic state resulting in increased deposition of connective tissue (Brack et al., 2007). The expression of myogenic regulatory factors (MRF) responsible for normal formation of new myotubes, such as MyoD, Myf5, myogenin, and Pax3/7, are activated by Wnt (Yokoyama and Asahara, 2011). However, in the event that these progenitor cells lose the ability to mediate repair, the muscle tissue is replaced by adipose and fibrotic tissue, which also appears to be a phenotype associated with WB (Laumonier and Menetrey, 2016). Our findings were not the first to detect changes in Wnt signaling due to WB. Others have shown that WB results in statistically significant differential expression of *WNT7A* (Zambonelli et al., 2014). Wnt7a is responsible for stimulating skeletal muscle growth and repair through the induction of satellite cells via the mTOR pathway (Bentzinger et al., 2014). In contrast, others have found that there is an increase in gene expression of the MRF’s however, they tend to vary depending on the lineage of the bird used (Velleman and Clark, 2015). This has led to a different understanding of the disease state which may not be entirely genetic or environmental but rather both, which is commonly referred to as epigenetic. It could be that environmental conditions stimulate pathways leading to genomic modifications, potentially resulting in phenotypic alterations.

Our working hypothesis, based on the ML analysis, is that the underlying mechanism resulting in fibrosis and hence, WB, is related to genetic regulation, possibly through NPCs, CCDC85A, and β-catenin. These activate the Wnt signaling pathway via TGF-β, mTOR and IGF-II pathways, potentially resulting in WB pathology. It is possible that a pattern of Mendelian inheritance does not result in direct causation of WB, but rather modifications that result in changes in the expression of genes such as histone modifications and DNA methylation.

Following damage or rapid growth, skeletal muscle satellite cells are failing to regenerate myoblasts and results in fibrotic scar tissue; overall, because stem cells are restricted to a limited number of divisions, we hypothesize satellite cells are being exhausted and eventually resulting in WB (Sacco et al., 2010). This is similar to the hypothesis presented by Daughtry et al. (2017), who thought that a disruption in satellite cell homeostasis was involved in muscle myopathies. Throughout the life of an organism, the number of satellite cells available for regeneration of cells decreases. For satellite cells, aging has been characterized by delayed activation and the inability to proliferate and differentiate. A decrease in the efficiency of Wnt, TGF and IGF signaling pathways has been shown to limit satellite cell proliferation and myoblast differentiation (Barton-Davis et al., 1998; Carlson et al., 2009). It is known that fast twitch muscle is the leading muscle type found in the PM of broilers. Fast twitch muscle has fewer satellite cells than those of slow twitch resulting in differences in the course of muscle regeneration (Collins and Partridge, 2005). Differences depicted in fast twitch fibers include the TGF-β expression pattern, early activation of the myogenic regulatory factors, and better regeneration efficiency (Zimowska et al., 2009, 2017). After injury, satellite cells are activated by expression of early myogenic regulatory factors, MyoD and Myf5. Next, late myogenic regulatory factors are expressed, which consist of myogenin and MRF4. Pax 3/7 are paired box transcription factors that directly and indirectly regulate myogeneic regulatory factors as skeletal muscle progenitor cells. Together these altered pathways are likely contributing to the development of the PM myopathy.

### Organismal Metabolic Influence – Liver Transcriptome

The liver was considered as a tissue of importance related to WB due to the vast array of metabolic functions including the responsibility to synthesize, metabolize and excrete many molecules (Zaefarian et al., 2019). In the bird, 11% of all protein synthesis occurs in the liver which are then transported via systemic circulation to other tissues (Denbow, 2000). Evaluation of the liver transcriptome dataset using IPA resulted in the identification of Skeletal and Muscular System Development and Function, Developmental Disorder, Hereditary Disorder (Figure 6), Connective Tissue Disorders, Hematological Disease, Hereditary Disorder (Figure 7) and Cell Cycle, Embryonic Development, Cellular Movement (Figure 8) network associations. Similarly when evaluating the PM through IPA analysis of differential gene expression data, connective tissue disorders, embryonic development and cell cycle pathways have previously been detected (Mutryn et al., 2015).

The association network Skeletal and Muscular System Development and Function, Developmental Disorder, Hereditary Disorder (Figure 6) depicts molecules involved in muscle function and development such as SGCG which protects and maintains the structure of muscle cells through the sarcoglycan protein. In mammals, mutations of this gene result in the loss of y-sarcoglycan protein and ultimatley muscle dystrophy and fibrosis (Heydemann et al., 2009). Disfunction in SGCG has been shown to result in enhanced TGF-β availability and therefore increased SMAD signaling leading to fibrosis (Heydemann et al., 2009). It is thought that proteins from this gene could be mediating their effect by regulating myostatin activity. Myostatin (MSTN), a family member of TGF-β, inhibits myoblast differentiation by repressing myogenic regulatory factors (Langley et al., 2002). MSTN prevents differentiation via the transcription factor SMAD3, which can be activated by both TGF-β and MSTN. Other molecules identified in IPA with changes in expression due to WB were JAKMIP2, which is invovlved in microtubule binding and CAPSL which is involved in calcium ion binding. This IPA pathway has also been detected when evaluating the PM of differential gene experssion between male and females birds with WB (Brothers et al., 2019).

CARD19 in this molecular network was also detected in MLP of WEKA as being able to predict normal or WB 93.333% using the cross-validation method (Figure 8 and Table 3). CARD proteins (caspase recruitment domain) are a domain of proteins which regulate apoptosis and inflammation (Jang et al., 2015). Studies evaluating CARD19 and its role in the IKKß and NF-kB pathway have been contradictory. Early data suggests CARD19 is a negative regulator of NF-kB, which is a transcription factors that signals IKK, however, a more recent study in mice suggests in the absence of CARD19 there was an increase in TNF-α which would subsequently increase IKKß and NF-kB (Rios et al., 2018). IKKß has been shown to decrease β-catenin activation which as previously mentioned is an activator canonical Wnt signaling (Lamberti et al., 2001; Staal, 2016).

Downstream TNF-α kinases IKKß and NF-kB play many roles in regulating physiological reactions including regulators of tuberous sclerosis complex (TSC), which repress the mechanistic Target of Rapamycin (mTOR) pathway (Lee et al., 2007). IKKß in association with TSC allows for the activation of mTOR. *MTOR* coordinates cell growth and is the major regulator of metabolic processes (Figure 7). Many factors are responsible for the activation of mTORC1 including Wnt signaling, growth factors, and TNF-α through interaction with TSC (Laplante and Sabatini, 2009). Activation of mTORC1 positively results in cell growth and proliferation through the activation of protein and lipid synthesis pathways. Disruptions in these pathways is associated with tumor development and fibrogenesis, and macrophage regulation. Ribosome biogenesis has been observed to be promoted through activation of mTORC1 by transcription of ribosomal RNA which can be observed as being downregulated in Figure 5 and Table 3 of the PM dataset (Mayer et al., 2004). Much of the IPA network association Connective Tissue Disorders, Hematological Disease, Hereditary Disorder invovles moleculues related to cell adhesion and cytoskeleton (Figure 7). Intersestingly, mTORC2 regulates cytoskeleton organization (Jacinto et al., 2004).

Cell Cycle, Embryonic Development, Cellular Movement (Figure 8) network association represents molecules in the cell cycle such as: ITCH, NEDD9, DOK5, and IGFBP2. ITCH in this molecular network was also detected in MLP of WEKA as being able to predict normal or WB 93.333% using the cross-validation method. The Itch protein encodes a member of the Nedd4 family ubiquitin ligases that targets specific proteins for lysosomal degradation. Itch plays a role in lymphoid cell differentiation and the regulation of immune response and pathways related to this protein include the TNF-α signaling pathway. NEDD9 plays a role in the TGF-β pathway and growth signals initiating cellular proliferation and has been identified as upregulated in hepatic fibrosis (Dooley et al., 2008). *DOK5* in humans is strongly expressed in muscle and is involved in the positive activation of MAPK and possibly insulin activation (Grimm et al., 2001; Cai et al., 2003). DOK5 has been identified as a membrane associated protein triggered by insulin-like growth factor binding protein-5 (IGFBP-5) for intracellular signaling resulting in pro-fibrotic effects and is thought to promote fibrosis (Yasuoka et al., 2014). IGFBP2 is an insulin-like growth factor binding protein involved in cellular signaling. While muscle is the primarily a site for glucose disposal, the avian liver does function to control muscle growth through the allocation of resources by regulating the birds nutritional balance. The muscle has a paracrine effect, whereas the liver has an endocrine role in circulating IGFs and IGFBPs. Signaling pathways in the liver play an important role in regulating many aspects of energy metabolism and cell cycle processes (Nguyen et al., 2014). Circulating molecules have been identified that play a role of signaling between the liver and muscle (Liu et al., 2013). Some signaling pathways are only responsive within the liver while others are only responsive in the muscle (proliferation and differentiation). Most of the IPA pathways identified resulted in an effect which would be detected within skeletal muscle. Liver and muscle are important tissues in understanding regulation of metabolic homeostasis, genes involved in glycogenesis, glycolysis, and lipogenesis are responsive in both liver and muscle even though the expression patterns are very different between the two tissues. It is not unexpected to observe effects of the liver on muscle development since the liver is the major location of protein, lipid, and carbohydrate metabolism supporting the rapid growth of broiler chickens.

Histological evaluation of WB has revealed multifocal degeneration and necrosis of fibers and accumulation of immune related cells such as macrophages, heterophils and lymphocytes (Sihvo et al., 2014; Trocino et al., 2015; Kuttappan et al., 2016). Affected areas have characteristics of fibrosis separating muscle fibers and thickening of the interstitium. Fibrosis has been characterized as hardening or scaring of tissue as a result of the accumulation of the extra cellular matrix proteins, including collagen and fibronectin eventually leading to loss of activity to the tissue (Wynn, 2008). Fibrosis has been detected in various tissues lung, liver, kidney and skeletal muscle, however, the mechanism resulting in fibrosis has been similar in tissues (Cisternas et al., 2014). Pathways detected through IPA of the liver and PM have indicated many molecules which directly and indirectly lead to tissue fibrosis. The fibrosis associated with WB may be driven by signal(s) originating in the liver or other tissues.

## Conclusion

In conclusion, using a machine learning approach, we were able to identify predictors that were able to accurately differentiate normal tissue from WB tissue using liver and PM transcriptomes from individual birds. Through the use of IPA, predictors from both PM and liver tissue identified gene networks associated with skeletal muscle disorders and other networks that could be associated with the development of WB. Given that gene expression data from both PM and liver transcriptomes were able to predict WB or normal tissue using select genes, with some redundancy between tissues, suggests that WB is the result of systematic disruptions in one or more regulatory pathways involving abnormal muscle development, deposition, or maintenance. The data herein suggests that WB phenotype could potentially be mediated through genes which ultimately result in the up- or down- regulation of pathways that are largely involved with metabolic regulation and basic cellular maintenance, such as Wnt and mTOR, respectively. In mammals, dysregulation in either of these canonical pathways has been shown to result in similar characteristics identified in WB and further investigation of these pathways in chickens exhibiting WB is warranted.

## Data Availability Statement

The datasets generated and analyzed for this study can be found in the NCBI-Gene Expression Omnibus database under Accession Series GSE144000: https://www.ncbi.nlm.nih.gov/geo/query/acc.cgi?acc=GSE144000.

## Ethics Statement

The animal study was reviewed and approved by the NC State Institutional Animal Care and Use Committee, 15-061-A, approved on 07/06/2015.

## Author Contributions

ML, KL, and CA conceived and designed the experiment. ML and KL performed the animal experiment. CA managed the RNA sequencing data collection and analysis. CP and BR analyzed the data. CP drafted the manuscript and it was revised and reviewed by BR, ML, KL, and CA.

## Conflict of Interest

The authors declare that the research was conducted in the absence of any commercial or financial relationships that could be construed as a potential conflict of interest.

## Footnotes

1 http://resources.qiagenbioinformatics.com/manuals/clcgenomicsworkbench/current/User_Manual.pdf

2 http://www.ingenuity.com
